# Differential impact of smoking on mortality and kidney transplantation among adult Men and Women undergoing dialysis

**DOI:** 10.1186/s12882-016-0311-x

**Published:** 2016-07-26

**Authors:** Austin G. Stack, Darya Yermak, David G. Roche, John P. Ferguson, Mohamed Elsayed, Waleed Mohammed, Liam F. Casserly, Stewart R. Walsh, Cornelius J. Cronin

**Affiliations:** 1 Departments of Nephrology and Medicine, University Hospital Limerick, Limerick, Ireland; 2 Graduate Entry Medical School, University of Limerick, Limerick, Ireland; 3 Health Research Institute (HRI), University of Limerick, Limerick, Limerick, Ireland; 4 Department of Medicine, Graduate Entry Medical School (GEMS), University of Limerick, Clinical Academic Liaison Building, St Nessans Rd, Limerick, Ireland

**Keywords:** Smoking, Transplantation, Mortality, End-stage kidney disease

## Abstract

**Background:**

The extent to which smoking contributes to adverse outcomes among men and women of all ages undergoing dialysis is uncertain. The objective of this study was to determine the differential impact of smoking on risks of mortality and kidney transplantation by age and by sex at dialysis initiation.

**Methods:**

We conducted a population-based cohort of incident U.S dialysis patients (*n* = 1, 220, 000) from 1995–2010. Age- and sex-specific mortality and kidney transplantation rates were determined for patients with and without a history of cardiovascular disease. Multivariable Cox regression evaluated relative hazard ratios (HR) for death and kidney transplantation at 2 years stratified by atherosclerotic condition, smoking status and age. Analyses were adjusted for demographic characteristics, non-cardiovascular conditions, laboratory variables, socioeconomic and lifestyle factors.

**Results:**

The average age was 62.8 (±15) years old, 54 % were male, and the majority was white. During 2-year follow-up, 40.5 % died and 5.7 % were transplanted. Age- and sex-specific mortality rates were significantly higher while transplantation rates were significantly lower for smokers with atherosclerotic conditions than non-smokers (*P* < 0.01). The adjusted mortality hazards were significantly higher for smokers with pre-existing coronary disease (HR 1.15, 95 % CI (1.11–1.18), stroke (HR 1.21, 1.16–1.27) and peripheral vascular disease (HR = 1.21, 1.17–1.25) compared to non-smokers without these conditions (HR 1.00, referent group). The magnitude of effect was greatest for younger patients than older patients. Contrastingly, the adjusted risks of kidney transplantation were significantly lower for smokers with coronary disease: (HR 0.60, 0.52–0.69), stroke; (HR 0.47, 0.37–0.60), and peripheral arterial disease (HR 0.55, 0.46–0.66) respectively compared to non-smokers without these conditions.

**Conclusions:**

We provide compelling evidence that smoking is associated with adverse clinical outcomes and reduced lifespans among dialysis patients of all ages and sexes. The adverse impact is greatest for younger men and women.

**Electronic supplementary material:**

The online version of this article (doi:10.1186/s12882-016-0311-x) contains supplementary material, which is available to authorized users.

## Background

A diagnosis of end-stage kidney disease (ESKD) is associated with reduced life expectancy with mortality rates of 20 % at 1-year and a 5-year survival of 30 % in US patients [1]. Atherosclerotic cardiovascular disease is a major contributor to mortality and accounts for over 60 % of all–causes of death [1]. The prevalence of coronary disease, stroke and peripheral arterial disease is high at dialysis initiation and each condition contributes independently to total and cardiovascular mortality [2–8]. The extent to which these conditions contribute to elevated death risk is determined by the effectiveness of cardiovascular treatment strategies and the degree to which existing atherosclerotic risk factors are controlled [7, 8, 9]. Despite these facts, it is surprising that only few studies exist that have addressed the clinical significance of smoking, an established modifiable risk factor, on clinical outcomes among patients with ESKD, [10–15]. The evidence to date would suggest that smoking among patients who require dialysis is associated with accelerated atherosclerosis and increased mortality. Furthermore, patients who smoke prior to kidney transplantation experience higher rates of graft loss, cardiovascular events and death [16–20].

Smoking is a well-established cause of excess mortality in the general population [21, 22]. It is a highly addictive behavior and remains the greatest risk factor for atherosclerotic disease in the developed world [23]. The extent to which smoking contributes to excess mortality in men and in women on dialysis with and without pre-existing atherosclerotic disease has not been fully explored. It is unclear for example whether continued smoking at dialysis initiation exerts greater impact on individuals with pre-existing coronary disease, peripheral vascular disease and stroke compared to patients without these conditions. It is equally uncertain whether and to what extent smoking alters the risk of receiving a kidney transplant among patients with pre-existing atherosclerotic conditions. Finally, there are few studies to our knowledge that have examined in detail the impact of smoking across age and sex groups. A better understanding of the hazards of smoking among these high-risk patients would help inform health policy and lend support for more aggressive smoking intervention strategies prior to and after the onset of renal replacement therapy.

The aim of this study was to 1) explore the relative contribution of smoking with mortality among new dialysis patients with and without atherosclerotic cardiovascular disease, and 2) to examine the association of smoking with likelihood of kidney transplantation taking into consideration differences in demographic, comorbid characteristics and 3) to determine whether associations with these outcomes differed between men and women and across representative age groups.

## Methods

### Data

Our hypotheses were tested in a historical prospective cohort of all incident dialysis patients from May 1995 and December 2008 using data from the U.S. Renal Data System [1]. This registry collects information on all patients who undergo dialysis or kidney transplantation in the United States. Data on demographic details, comorbid conditions, lifestyle factors, laboratory values and dates of dialysis initiation were captured on the Center for Medicare and Medicaid Services (CMS) Medical Evidence Form, which is completed for all patients at dialysis initiation [24]. Smoking status was recorded as current tobacco use at dialysis initiation. The following variables were included for our analyses: age, gender, race, the presence or absence of coronary artery disease, peripheral arterial disease, stroke, diabetes, hypertension, congestive heart failure, chronic lung disease, malignancy, alcohol use, drug dependence, inability to ambulate and transfer independently, current employment status, and pre-dialysis use of erythropoietin. Laboratory data included serum creatinine measured at or prior to dialysis initiation and serum albumin. Body mass index (BMI) was estimated from recorded weight and height measurements and residual renal function at dialysis initiation was estimated from estimated glomerular filtration rate (eGFR) using the Chronic Kidney Disease Epidemiology Collaboration (CKD-EPI) [25].

### Cohort assembly

An observational cohort was assembled using the standard analysis files (SAF) of the US Renal Data System. The Medical Evidence File (SAF.Medevid, n =1,753,496), Mortality file (SAF.Patients, *n* = 2,377,166), and the Treatment history file (SAF.Rxhist90, *n* = 13,038,727) were merged by unique patient identity number. Patients were excluded if; there was unknown data on race, sex, cause of ESKD; date of first treatment occurred before May 1995; medical evidence forms were completed prior to 5/1/1995 or after 12/31/2010, and if age < 18 and > 100 years and leaving a sample of 1,445,980 patients. In addition, we further restricted the sample to the period 5/1/1995- 12/31/2008 to ensure a minimum of 2 years follow-up on all participants leaving 1,220, 000 patients in the final study cohort.

### Statistical analysis

The prevalence and distribution of coronary disease, peripheral arterial disease and stroke, by smoking status was compared for the entire incident population. Comparisons between groups were conducted using the chi-square and t-tests. Multivariate logistic regression explored the independent relationships of demographic, clinical, laboratory, lifestyle variables and elements of pre-dialysis care with current smoking status and associations were represented by adjusted odds-ratios (AOR) and 95 % confidence intervals (CI).

Two-year mortality rates were calculated for subjects with and without each atherosclerotic condition stratified by smoking status. Poisson regression compared mortality rates while Cox regression modeled survival times for each condition stratified by smoking status. The study start date was defined as the date of first regular dialysis and patients were followed until the earliest of death, kidney transplantation, loss to follow-up, end of 2 years, or December 2010. Unadjusted and adjusted hazard ratios (HR) were calculated and models of increased complexity were constructed to explore the conjoint associations of each condition and smoking status with mortality. In the final model, we adjusted for a comprehensive set of clinical, laboratory, lifestyle, functional, and pre-dialysis factors. Interactions between smoking and each atherosclerotic condition were tested for in the multivariable Cox model with a *P*-value <0.01 determined as significant. In addition, we specifically tested for effect modification of age on the association of smoking status with each primary outcome. Stratum-specific hazards ratios were determined for each subgroup for significant interactions. The proportionality of the Cox model was tested by assessing Shoenfield residuals and time-covariate interactions. Finally, all analyses were repeated with date of first kidney transplant as the major outcome and death as a censoring variable. Statistical analysis was performed using SAS statistical software (version 9.3, SAS institute, Cary, NC, USA).

## Results

### Baseline characteristics

The study cohort included 1, 220, 000 individuals who commenced dialysis between May 1995 and December 2008 and followed until December 2010. The mean age of subjects was 62.7 years, 54 % were male, and the majority was white. Subjects with atherosclerotic conditions were on average younger, male, black, diabetic, had higher prevalence of chronic lung disease, drug and alcohol use, and a higher percentage of patients reporting difficulty with walking and transferring independently (Tables 1 and 2). Younger age, non –white race, most cardiovascular conditions, drug and alcohol dependence were significantly associated with smoking at dialysis initiation (Additional file 1: Table S1). Patients age 18 to 40 years and 50 to 60 years were between 8–11 times more likely to smoke compared to those age > 80 years. Drug and alcohol dependence were also significantly associated with smoking while employment was inversely related. The model had good discrimination with a C-statistic of 78 %.

**Table 1:** Baseline Characteristics of Overall Population and of Patients with and without Coronary Disease by Smoking Status

			Coronary Artery Disease		No Coronary Disease	
		Overall	Smoking Status		Smoking Status	
			Yes	No	Yes	No
			(*n* = 19,271)	(*n* = 295,192)	49,459	856,020
Demographics						
	Age (years)	62.8 (15.3)	63 (11.3)	69.6 (11.2)*	54.3 (13.8)	60.9 (15.9)*
Gender (%)						
	Male	54.3	66.5	57.9*	63.3	52.3*
Race (%)						
	White	65.4	77.3	77.6	59.9	61.2*
	Black	29.7	20.3	18.4*	37.4	33.4*
	Asian	3.8	1.1	2.9*	1.5	4.3*
	Native American	1.11	1.3	1*	1.2	1.2
Hispanic (%)						
	Hispanic	7.8	2.8	6.6*	3	8.6*
Comorbid Conditions (%)						
	Diabetes (cause of ESKD)^a^	45.3	45.3	53.5*	33.3	43.2*
	Diabetes (as comorbid condition)	52.8	55.3	62.9*	40.4	50*
	Hypertension	79.4	88.8	83.8*	84	77.4*
	Heart Failure	33.1	57.3	59.2*	24.2	24.1
	Coronary Disease	25.8				
	Stroke	9.5	20.8	16.2*	8.9	7*
	Peripheral Arterial Disease	14.7	43.2	30.8*	13.4	8.5*
	Chronic lung disease	8.2	41.2	13*	18.7	5.1*
	Malignancy	6.4	7.6	7.3	6.4	6
	Body Mass Index (kg/m^2^)	27.5 (7.3)	26 (6.7)	27.3 (6.9)*	26.1 (7.1)	27.7 (7.5)*
Lifestyle factors (%)						
	Alcohol Dependence	1.5	6.1	0.7*	10.6	1.2*
	Drug dependence	1.2	2.7	0.3*	8.6	1*
Functional Status (%)						
	Inability to walk independently	5.2	8.2	8	4.4	4.2
	Inability to transfer independently	2.1	2.6	3.2*	1.4	1.8*
Employment Status (%)						
	Full time employment	8.2	4.5	3.4*	9.7	9.8
	Part time employment	1.8	1.4	1.1*	2.2	2*
	Unemployed	19.8	16.6	11.5*	31.1	22*
	Homemaker	4.8	3.5	5.2*	3	4.9*
	Retired from age	39.8	39.1	55.6*	20.8	35.4*
	Retired from disability	19.4	29.7	18.9*	26.5	18.9*
	Medical leave of absence	3.0	2.4	1.4*	4.3	3.5*
	Student	0.29	0.03	0.02	0.2	0.4*
	Other	3.0	2.6	2.7	2.2	3.1*
Laboratory Variables						
	Serum Creatinine (μmol/L)	654.2 (309.4)	601.7 (251.4)	560.9 (226.4)*	726.3 (351.9)	681.2 (330.4)*
	eGFR^b^ (ml/min/1.73 m^2^)	8.6 (3.8)	8.8 (3.7)	8.8 (3.6)	8.0 (3.8)	7.9 (3.6)*
	Albumin (g/L)	31 (7.0)	31 (6.6)	31.3 (6.4)*	3.1 (0.7)	3.1 (0.7)*
Pre-ESRD Care (%)						
	Erythropoietin use	29.0	27.4	32.2*	23.2	28.3*
Dialysis Modality at day 90 (%)						
	Peritoneal Dialysis	7.0	6.5	5.3*	7.9	7.5
	Hemodialysis	80.4	79.6	78.7	81.4	80.9

All values are reported as % or means with standard deviation

^a^ESKD: end-stage kidney disease

^b^Glomerular filtration rate (ml/min per 1.73 m^2^) was based on the Chronic Kidney Disease Epidemiology Collaboration [25]

*P < 0.001 for all bivariate comparisons

58 patients from the original cohort of 1, 220, 000 had missing data leaving 1, 219, 942 patients

**Table 2:** Characteristics of patients with and without Stroke and Peripheral Arterial Disease by Smoking Status at dialysis initiation

		Stroke		No Stroke		Peripheral Arterial Disease		No Peripheral Arterial Disease	
		Smoking Status		Smoking Status		Smoking Status		Smoking Status	
		Yes	No	Yes	No	Yes	No	Yes	No
		(*n* = 8,384)	(*n* = 107, 806)	60,339	1,043,406	(*n* = 14, 960)	(*n* = 163, 846)	53,763	987,379
Demographics									
	Age (years)	61.9 (11.7)	68.4 (12)*	56 (13.8)	62.6 (15.5)*	63.1 (11.3)	68.1 (11.9)*	55 (13.8)	62.3 (15.7)*
Gender (%)									
	Male	63.3	52.5*	64.3	53.9*	65.4	57.9*	63.9	53.1*
Race (%)									
	White	69.1	66.2*	64.2	65.3*	79	76.2*	60.9	63.6*
	Black	28.6	29.5	33.1	29.6*	18.8	20.3*	36.4	31.1*
	Asian	1.1	3.3*	1.4	4*	0.8	2.1*	1.6	4.2*
	Native American	1.3	0.9	1.2	1.1	1.4	1.4	1.2	1.1
Hispanic (%)									
	Hispanic	2.3	5.8*	3.1	8.3*	2.8	7.2*	3	8.2*
Comorbid Conditions (%)									
	Diabetes (cause of ESKD)^a^	44	52.1*	35.6	45.2*	48.5	59.4*	33.4	43.5*
	Diabetes (comorbid condition)	54	61.8*	43.3	52.4*	57.9	68*	40.9	50.9*
	Hypertension	91.1	87.2*	84.5	78.2*	89.7	85.8*	84.1	77.9*
	Heart Failure	45	46.8	31.9	31.7	52	56.9*	28.3	29.1*
	Coronary Disease	47.7	44.3*	25.3	23.7*	55.6	55.4	20.4	20.7
	Stroke					22.4	19.5*	9.4	7.7*
	Peripheral Arterial Disease	40	29.6*	19.2	12.6*				
	Chronic lung disease	35.7	11.9*	23.5	6.7*	43.6	14.2*	19.8	6*
	Malignancy	8	7.3	6.5	6.2	7.4	6.1*	6.5	6.4
	Body Mass Index (kg/m^2^)	25.5 (6.6)	26.8 (6.8)*	26.2 (7)	27.7 (7.4)*	25.6 (6.7)	27.4 (7.3)*	26.2 (7)	27.6 (7.4)*
Lifestyle factors (%)									
	Alcohol Dependence	8.2	1.0*	9.5	1.1*	6.1	0.8*	10.3	1.1*
	Drug dependence	4.2	0.6*	7.4	0.8*	2.7	0.4*	8.2	0.9*
Functional Status (%)									
	Inability to walk independently	11.5	13.6*	4.6	4.3*	11.3	11.9	3.8	4
	Inability to transfer independently	4.3	6.5*	1.4	1.7*	3.5	4.8*	1.2	1.7*
Employment Status (%)									
	Full time employment	3	2.2*	9	8.8	4	3.1*	9.4	9*
	Part time employment	0.8	0.7	2.2	1.9*	1.4	1*	2.2	1.9*
	Unemployed	19.6	14.1*	28.1	19.9*	16.1	13.3*	30.1	20.3*
	Homemaker	3.6	5*	3.1	4.9*	3.6	4.9*	3	4.9*
	Retired from age	35.8	51.2*	24.6	39.5*	39.3	51.3*	22.2	38.8*
	Retired from disability	33	23.3*	26.6	18.5*	31.2	22.3*	26.4	18.4*
	Medical leave of absence	1.9	1.2*	4	3.1*	2	1.3*	4.3	3.2*
	Student	0.05	0.05	0.1	0.3*	0.03	0.04	0.2	0.3*
	Other	2.3	2.4	2.3	3.1*	2.4	2.7	2.3	3.1*
Laboratory Variables									
	Serum Creatinine (μmol/L)	616 (259)	580 (244)*	702 (340)	658 (317)*	594 (248)	564 (228)*	719 (347)	665 (321)*
	eGFR^b^ (ml/min/1.73 m^2^)	8.7 (3.7)	8.6 (3.6)	8.1 (3.8)	8.1 (3.7)*	8.9 (3.8)	8.9 (3.7)	8 (3.8)	8 (3.6)
	Albumin (g/L)	30.7 (6.7)	30.9 (6.6)	3.1 (0.7)	3.1 (0.7)*	30.6 (6.8)	30.7 (6.6)	3.1 (0.7)	3.2 (0.7)*
Pre-ESRD Care (%)									
	Erythropoietin use pre-dialysis	25.6	30.1*	24.2	29.2*	28.2	32.7*	23.3	28.7*
Dialysis Modality at day 90 (%)									
	Peritoneal Dialysis	5.9	4.5*	7.7	7.2*	6.2	4.9*	7.8	7.3*
	Hemodialysis	80.4	79.8	81	80.4*	79.5	79.2	81.3	80.6*

All values are reported as % or means with standard deviation

^a^ESKD: end-stage kidney disease

^b^Glomerular filtration rate (ml/min per 1.73 m^2^) was based on the Chronic Kidney Disease Epidemiology Collaboration [25]

*P < 0.001 for all bivariate comparisons

### Death rates among patients with atherosclerotic disease at dialysis initiation by smoking status

**Fig. 1:**
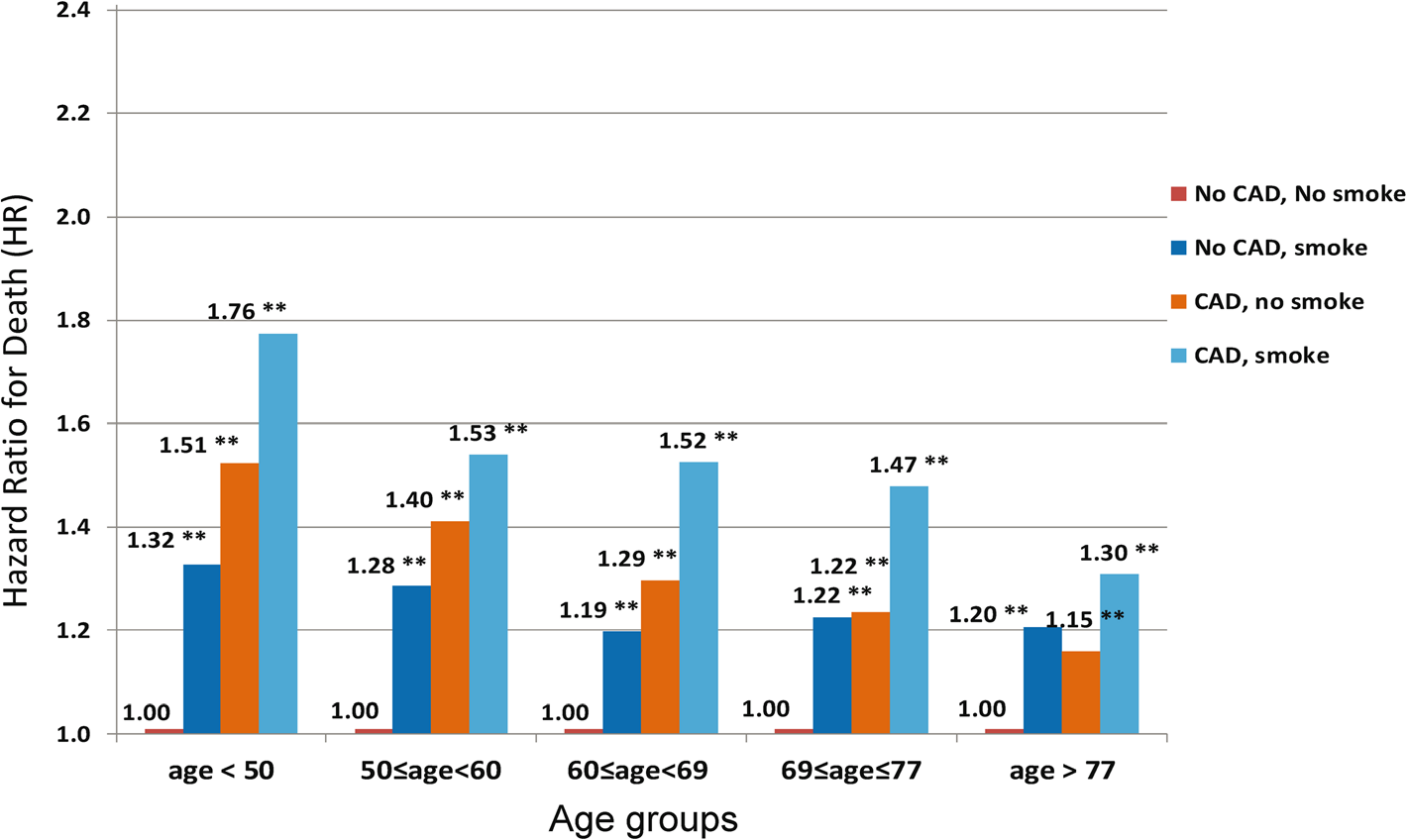
Hazard Ratios for Death for groups stratified by Coronary Disease, smoking status and age. P value for each group versus referent (no disease, non-smoker), **P < 0.001. Model adjusted for demographic characteristics (sex and race), clinical conditions (diabetes, hypertension, peripheral vascular disease, cerebrovascular disease, heart failure, pulmonary disease, cancer, AIDS, body mass index), lifestyle and functional factors (difficulty in walking and transfers, alcohol use, and employment status), erythropoietin use pre-dialysis, and laboratory factors (serum albumin, eGFR at dialysis initiation). Age was modeled in quintiles

The overall mortality at 2 years was significantly higher in smokers with pre-existing atherosclerotic conditions than non-smokers. Age-specific mortality rates for coronary disease, stroke and peripheral arterial disease were significantly higher for smokers than non-smokers and this pattern was consistent for men and women (Additional file 2: Figure S1a-c, Additional file 3: Figure S2a-c). The conjoint associations of coronary disease and smoking status with mortality are illustrated in Table 3. The interaction between smoking and coronary disease with 2-year mortality was significant (*P* <0.01). Compared to non-smokers without coronary disease (referent HR 1.00), the demographic-adjusted HR for death was significantly higher for smokers without disease HR 1.26 (1.24–1.28) and greatest for smokers with coronary disease HR 1.50 (1.47–1.53). In the fully adjusted model, patients with coronary disease who continued to smoke experienced the greatest mortality risks HR 1.14 (1.10–1.17). On further examination, we found that the interaction between coronary disease and smoking with mortality was further modified by age and this was confirmed when a 3-way interaction term with age was included in the model. The HR of smoking on mortality was significantly greater for younger than older patients (Fig. 1), and the magnitude of the effect increased progressively for younger patients.

**Table 3:** Hazards Ratios (HR) for Death for each Atherosclerotic Condition by Smoking Status among new dialysis Patients^a^

	With Coronary Disease		Without Coronary Disease	
Group	Smokers	Non-smokers	Smokers	Non-smokers
	HR (95 % CI)	HR (95 % CI)	HR (95 % CI)	HR (95 % CI)
				Referent
Unadjusted	1.55 (1.52–1.58)	1.65 (1.64–1.66)	0.96 (0.95–0.98)	1.00
Demographic-adjusted^b^	1.50 (1.47–1.53)	1.25 (1.25–1.26)	1.26 (1.24–1.28)	1.00
Adjusted for all covariates^c^	1.14 (1.11–1.17)	1.10 (1.09–1.11)	1.09 (1.07–1.12)	1.00
	With Stroke		Without Stroke	
	Smokers	Non-smokers	Smokers	Non-smokers
Group	HR (95 % CI)	HR (95 % CI)	HR (95 % CI)	HR (95 % CI)
				Referent
Unadjusted	1.41 (1.37–1.46)	1.54 (1.52–1.55)	0.96 (0.95–0.98)	1.00
Demographic-adjusted^b^	1.53 (1.49–1.58)	1.32 (1.31–1.33)	1.25 (1.24–1.27)	1.00
Adjusted for all covariates^c^	1.22 (1.19–1.25)	1.18 (1.17–1.20)	1.08 (1.06–1.10)	1.00
	With Peripheral Arterial Disease		Without Peripheral Arterial Disease	
	Smokers	Non-smokers	Smokers	Non-smokers
Group	HR (95 % CI)	HR (95 % CI)	HR (95 % CI)	HR (95 % CI)
				Referent
Unadjusted	1.55 (1.52–1.59)	1.59 (1.57–1.60)	0.92 (0.91–0.93)	1.00
Demographic-adjusted^b^	1.56 (1.52–1.59)	1.33 (1.32–1.34)	1.23 (1.22–2.25)	1.00
Adjusted for all covariates^c^	1.22 (1.18–1.26)	1.14 (1.13–1.16)	1.07 (1.05–1.10)	1.00

^a^Hazards Ratio (HR) and 95 % confidence interval

^b^Demographic variables of age, gender and race

^c^Final multivariable model adjusted for demographic characteristics (age, sex and race), clinical conditions (diabetes, hypertension, coronary disease, peripheral vascular disease, cerebrovascular disease, heart failure, pulmonary disease, cancer, AIDS, body mass index), lifestyle and functional factors (difficulty in walking and transfers, alcohol use), socioeconomic factors (employment status), erythropoietin use pre-dialysis, and laboratory factors (serum albumin, eGFR at dialysis initiation). Note the atherosclerotic condition was not included in the adjusted analysis, if it was the primary stratifying variable

**Fig. 2:**
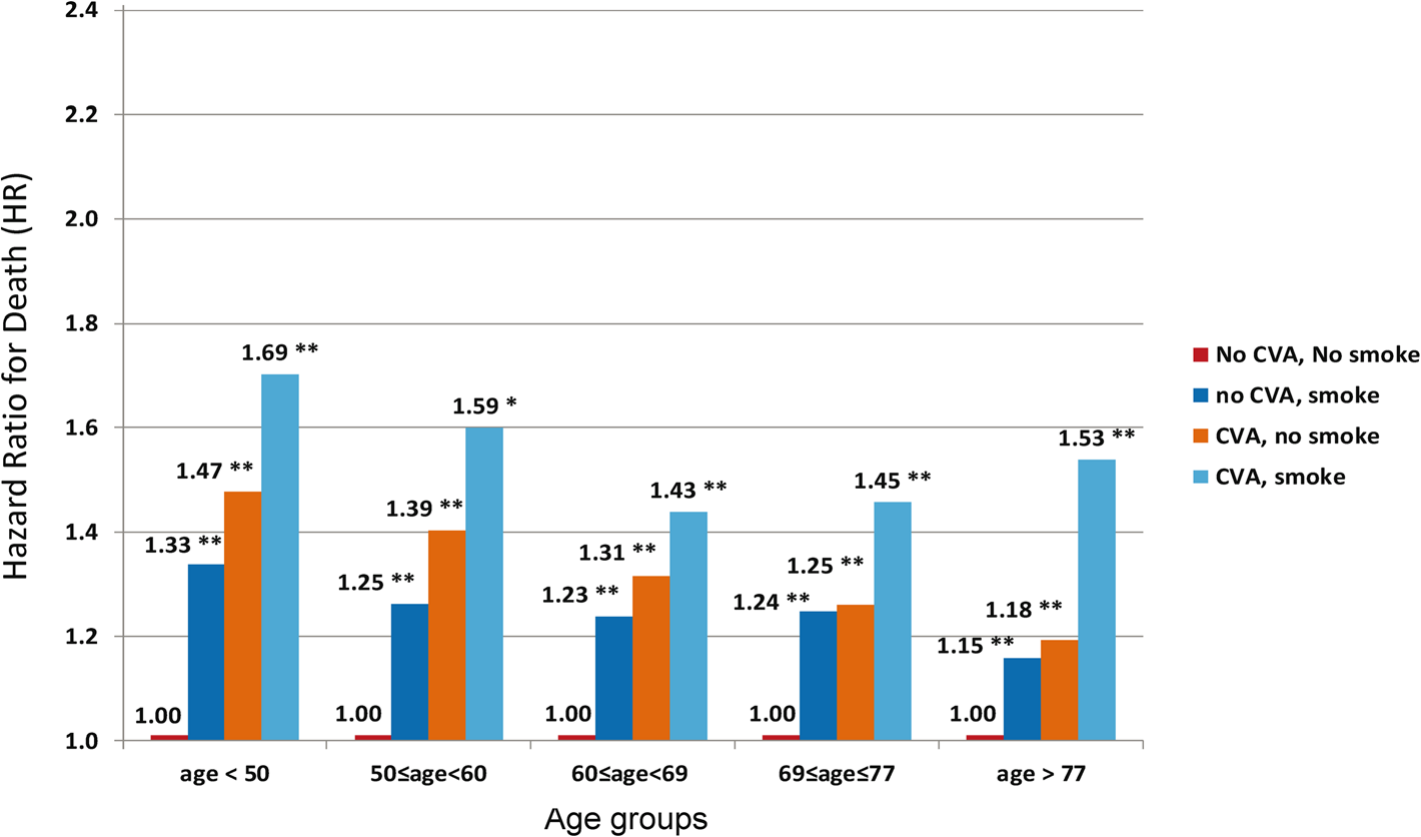
Hazard Ratios for Death for groups stratified by Stroke, smoking status and age. P value for each group versus referent (no disease, non-smoker), **P < 0.001. P value for each group versus referent (no disease, non-smoker), **P < 0.001. Model adjusted for demographic characteristics (sex and race), clinical conditions (diabetes, hypertension, peripheral vascular disease, coronary disease, heart failure, pulmonary disease, cancer, AIDS, body mass index), lifestyle and functional factors (difficulty in walking and transfers, alcohol use, and employment status), erythropoietin use pre-dialysis, and laboratory factors (serum albumin, eGFR at dialysis initiation). Age was modeled in quintiles

**Fig. 3:**
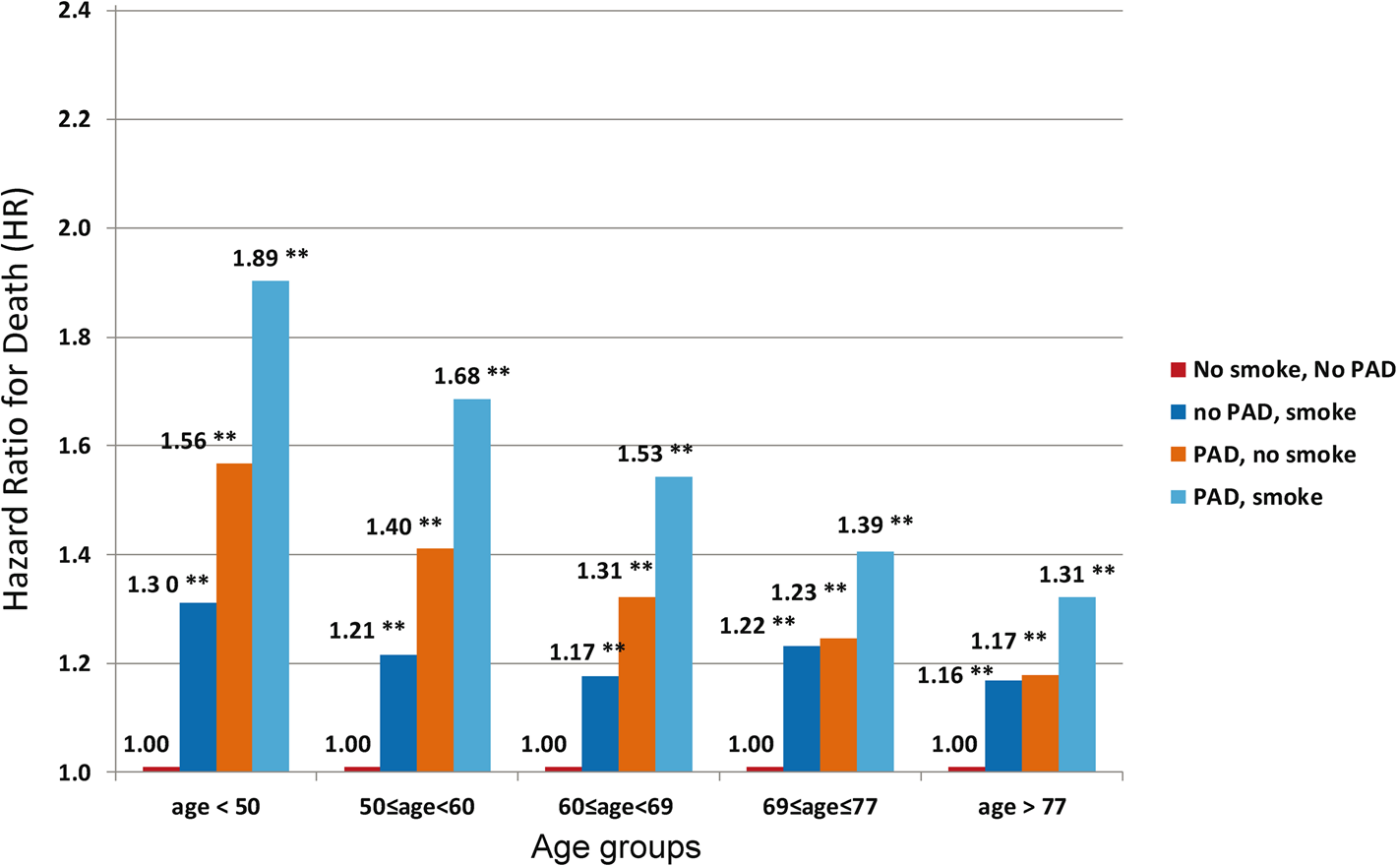
Hazard Ratios for Death for groups stratified by Peripheral Arterial Disease, smoking status and age. P value for each group versus referent (no disease, non-smoker), **P < 0.001. P value for each group versus referent (no disease, non-smoker), **P < 0.001. Model adjusted for demographic characteristics (sex and race), clinical conditions (diabetes, hypertension, coronary disease, cerebrovascular disease, heart failure, pulmonary disease, cancer, AIDS, body mass index), lifestyle and functional factors (difficulty in walking and transfers, alcohol use, and employment status), erythropoietin use pre-dialysis, and laboratory factors (serum albumin, eGFR at dialysis initiation). Age was modeled in quintiles

For stroke patients, the mortality pattern was similar to those with coronary disease (*P* < 0.01 for the interaction). Mortality risks were significantly higher for stroke patients who continued to smoke compared to those who did not with adjustment for demographic characteristics [HR 1.53 (1.49–1.58) versus HR 1.32 (1.31–1.33)] versus the referent category (HR = 1.00). The association was attenuated but remained significant in the fully adjusted model. Similarly, the relationship of smoking status with mortality among stroke patients also varied according to age (Fig. 2) with the greatest hazards of death present for younger patients. For patients with peripheral arterial disease, the pattern of association was similar to those described above although the interaction term was not significant (Fig. 3).

### Transplant rates among patients with atherosclerotic disease at dialysis initiation by smoking status

Age-specific transplant rates for coronary disease, stroke and peripheral vascular diseases were significantly lower for patients who smoked than those who did not and this pattern was consistent for men and women (Additional file 4: Figure S3a-3c, Additional file 5: Figure S4a-4c). For patients with coronary disease, transplant rates were significantly lower for younger patients who smoked than those who did not. For example among men age 20–40, transplantation rates were 45 per 1000 person-years for smokers versus 88 per 1000 person years for non-smokers. With advancing age, this disparity narrowed as overall rates between groups decreased significantly.

The associations of coronary disease with transplantation for smokers and non-smokers are shown in Table 4. Compared to the referent (non-smokers without coronary disease), the demographic adjusted-HR for transplantation was lowest among patients with coronary disease who continued to smoke, HR 0.36 (0.33–0.40). In the fully adjusted model, patients with coronary disease who continued to smoke experienced the lowest risk of transplantation HR 0.61 (0.53–0.69). The risk associations for patients with stroke or peripheral vascular disease according to smoking status followed a similar pattern. For stroke patients, transplantation risks were significantly poorer among those who continued to smoke compared to those who did not adjusting for demographic factors HR 0.50 (0.40-0.63) versus the referent category. Similarly, for patients with peripheral arterial disease, this pattern of association was virtually identical HR 0.57 (0.48–0.67).

**Table 4:** Hazards Ratios (HR) for Kidney Transplantation for each Atherosclerotic Condition by Smoking Status among new dialysis Patients^a^

	With Coronary Disease		Without Coronary Disease	
Group	Smokers	Non-smokers	Smokers	Non-smokers
	HR (95 % CI)	HR (95 % CI)	HR (95 % CI)	HR (95 % CI)
				Referent
Unadjusted	0.31 (0.28–0.34)	0.35 (0.34–0.36)	0.78 (0.76–0.81)	1.00
Demographic-adjusted^b^	0.36 (0.33–0.40)	0.59 (0.57–0.61)	0.60 (0.57–0.62)	1.00
Adjusted for all covariates^c^	0.61 (0.53–0.69)	0.76 (0.73–0.79)	0.74 (0.70–0.78)	1.00
	With Stroke		Without Stroke	
	Smokers	Non-smokers	Smokers	Non-smokers
	HR (95 % CI)	HR (95 % CI)	HR (95 % CI)	HR (95 % CI)
				Referent
Unadjusted	0.31 (0.26–0.36)	0.31 (0.30–0.33)	0.79 (0.76–0.82)	1.00
Demographic-adjusted^b^	0.34 (0.29–0.40)	0.50 (0.48–0.52)	0.59 (0.57–0.61)	1.00
Adjusted for all covariates^c^	0.50 (0.40–0.63)	0.64 (0.59–0.68)	0.75 (0.71–0.79)	1.00
	With Peripheral Arterial Disease		Without Peripheral Arterial Disease	
	Smokers	Non-smokers	Smokers	Non-smokers
	HR (95 % CI)	HR (95 % CI)	HR (95 % CI)	HR (95 % CI)
				Referent
Unadjusted	0.32 (0.29–0.36)	0.32 (0.31–0.33)	0.81 (0.78–0.84)	1.00
Demographic-adjusted^b^	0.35 (0.31–0.39)	0.46 (0.44–0.48)	0.59 (0.57–0.62)	1.00
Adjusted for all covariates^c^	0.57 (0.48–0.67)	0.66 (0.62–0.70)	0.74 (0.70–0.78)	1.00

^a^Hazard Ratios for first kidney transplant (HR) and 95 % confidence intervals

^b^Demographic variables of age, gender and race

^c^Final multivariable model adjusted for demographic characteristics (age, sex and race), clinical conditions (diabetes, hypertension, coronary disease, peripheral vascular disease, cerebrovascular disease, heart failure, pulmonary disease, cancer, AIDS, body mass index), lifestyle and functional factors (difficulty in walking and transfers, alcohol use), socioeconomic factors (employment status), erythropoietin use pre-dialysis, and laboratory factors (serum albumin, eGFR at dialysis initiation). Note the atherosclerotic condition was not included in the adjusted analysis, if it was the primary stratifying variable

## Discussion

In this national study, we report associations of smoking with higher rates of death and lower rates of kidney transplantation in a contemporary cohort of dialysis patients. Smoking at dialysis initiation was associated with excess mortality for both men and women with and without pre-existing cardiovascular disease. Although these risks were present for patients of all ages, they were greatest among younger patients who started dialysis and continued into old age. Although continued smoking was associated with several known mortality predictors, adjustment for these factors did not abolish the risk. Smoking was equally detrimental to kidney transplantation among men and women with atherosclerotic cardiovascular disease. These findings demonstrate that smoking remains a substantial, yet modifiable, contributor to overall poor outcomes for men and women of all ages at dialysis initiation both directly from high death rates and indirectly from low rates of kidney transplantation.

The present study sheds new light on the mortality risks of continued smoking among those with and without atherosclerotic disease at dialysis initiation. These risks were substantial such that continued smoking conferred on average an additional 25 % higher death risk for patients with stroke, coronary disease or peripheral arterial disease. The following observations are noteworthy and highlight the importance of smoking as a major mortality risk factor in dialysis cohorts. *First,* smoking increased the risk of death for all patients with newly diagnosed ESKD regardless of disease status. *Second*, the highest risk groups in each category were those patients who had known coronary disease, stroke and peripheral arterial disease and who continued to smoke. *Third,* the pattern of risk was similar for men and women but was greatest for younger dialysis patients and continued right into old age without exception. These findings suggest that patients with atherosclerotic disease and who continue to smoke following dialysis experience a greatly magnified risk of death beyond the risk that associated with dialysis and co-existing comorbidity.

What is striking from this analysis is the extent to which smoking increased mortality risks across all representative age and sex groups, an observation which has not been previously demonstrated [13]. From the youngest to the oldest patients, in men and in women, mortality rates were significantly higher in smokers than non-smokers and the pattern was consistent for each major condition. The negative impact of smoking on mortality was present in young adults age 18–40, and continued right into older age > 70 years without exception. Our analysis uncovered a very strong age effect in that the mortality impact of smoking was greatest among younger patients than older patients. Although this is not surprising as younger patients are less likely to have competing mortality risks compared with older patients, it nevertheless reinforces the concept that smoking remains a substantial modifiable factor among young patients who reach ESKD. Equally important, the elevated mortality associated with smoking was carried into old age and the magnitude of the risk was similar to other established mortality predictors including malnutrition and chronic lung disease. These findings would suggest that smoking cessation programs should be pursued for all smokers, irrespective of age, who approach ESKD.

A key objective of this study was to define the association of smoking with hazards of kidney transplantation. In this analysis, we provide evidence that smoking was associated with lower likelihood of transplantation but disproportionately more so for those with atherosclerotic conditions. Smoking may reduce the likelihood of kidney transplantation either due to worsening of existing comorbidity or due to the development of de-novo smoking-related comorbidity while on dialysis. In support, we found that smokers at dialysis initiation were far more likely to have several comorbid medical conditions and adverse lifestyle profiles. Our findings extend the observations of Sandhu et al. who found that smoking was associated with reduced rates of kidney transplantation [26]. Not only did we confirm their observations, we provide further evidence that the negative impact of smoking is substantial, worse among those with atherosclerotic disease, and extends to almost all demographic groups and did not abate following multivariable adjustment. The impact of smoking on kidney transplant rates among young adult smokers with atherosclerotic disease is noteworthy and concerning given that young patients are generally considered to have the greatest opportunity of receiving a kidney transplant. For example, the rates of kidney transplantation among young men with coronary disease were reduced from 88 to 45 per 1000 person-years of follow up and similar patterns were observed for men with stroke and peripheral arterial disease. These data provide further compelling evidence that smoking is associated with reduced kidney transplantation rates among those who may benefit most.

There are limited studies in the literature that have explored the relationship of smoking with clinical outcomes among incident dialysis cohorts. While, previous studies have demonstrated that smoking is associated with adverse clinical outcomes, most of these have considered smoking as an adjustment variable rather than the primary exposure [10–15]. Furthermore, to our knowledge, only a single published study has explored risks of smoking among dialysis patients with pre-existing atherosclerotic disease [10].

The current body of evidence would suggest that tobacco use is an independent predictor of all-cause and infection-related mortality [11, 13–15] and de-novo vascular disease [10]. However, it has been unclear until now the extent to which smoking contributes to death and transplantation in men and in women with atherosclerotic disease and across different age categories. Defining the magnitude and extent of these risks provides further compelling evidence to physicians, professional bodies and to policy makers that smoking is detrimental to all patients following dialysis initiation.

This observational study is not without limitations. The classification of smoker status was based on the Medical Evidence record at dialysis initiation, which may underreport this behavior [27]. Misclassification bias would tend to underestimate the true magnitude of exposure-outcome associations. Furthermore, our analysis did not capture the number of cigarettes smoked or pack-years and consequently we were unable to assess the duration of exposure or a gradient effect. We submit, however, that the present study is no substitute for well-designed prospective studies that could accurately confirm smoking status, and categorize the duration and pack years. We also recognize the limitations of observational studies in identifying cause and effect associations. Despite these limitations, this study had several strengths that are worthy of mention. Its large sample size and the representativeness of the US dialysis population allow excellent generalizability to all age and sex subgroups. The US registry captured information on a large number of demographic, clinical, laboratory and lifestyle characteristics, which facilitated a comprehensive adjustment for confounding as well as the opportunity to explore effect sizes in men and in women and cross all age groups.

## Conclusions

The present study highlights the detrimental effect of smoking on the risk of death and kidney transplantation among new patients with and without pre-existing atherosclerotic conditions. Smoking contributed significantly to higher death rates and lower rates of kidney transplantation for men and women of all ages. These risks were substantial, greatest for younger patients, and largely independent of known mortality predictors. Although, it might be expected that smokers would do worse, the magnitude of the impact was far greater than we anticipated especially among younger patients. This study highlights the need for greater recognition of smoking as a mortality risk factor and risk amplifier for adverse outcomes for patients who require dialysis. Our study reaffirms a key message for all healthcare providers that smoking cessation strategies should be relentlessly pursued in all patients across the spectrum of CKD including those who reach end-stage kidney disease.

## Abbreviations

95 % CI, 95 % confidence intervals; AOR, Adjusted odds ratio; CKD-EPI, Chronic Kidney Disease Epidemiology Collaboration; CMS, Center for Medicare and Medicaid Services; eGFR, Glomerular filtration rate; ESKD, End-stage kidney disease; HR, Hazard ratio; Native Am, Native American/Alaskan Native; SAF, Standard analysis files; USRDS, United States Renal Data System.

## Additional files

Additional file 1: Table S1. Factors associated with the presence of Smoking at dialysis onset among new Dialysis Patients^1^. Multivariable analyses of factors associated with smoking at dialysis initiation. Model included demographic, clinical, biochemical, lifestyle, employment and functional status indicators measured at dialysis onset. C-statistic 78 %, ^2^ 95 % confidence intervals. (DOC 59 kb) Additional file 2: Figure S1a-c. Age-specific mortality rates for Coronary disease, Peripheral Arterial Disease and Stroke for Men by smoking status. P–value for differences between smokers and non-smokers ***P* < 0.01, **P* < 0.05. (ZIP 665 kb) Additional file 3: Figure S2a-c. Age-specific mortality rates for Coronary disease, Peripheral Arterial Disease and Stroke for Women by smoking status. P–value for differences between smokers and non-smokers ***P* < 0.01, **P* < 0.05. (ZIP 642 kb) Additional file 4: Figure S3a-c. Age-specific transplantation rates for Coronary disease, Peripheral Arterial Disease and Stroke for Men by smoking status. P–value for differences between smokers and non-smokers ***P* < 0.01, **P* < 0.05. (ZIP 586 kb) Additional file 5: Figure S4a-c. Age-specific transplantation rates for Coronary disease, Peripheral Arterial Disease and Stroke for Women by smoking status. P–value for differences between smokers and non-smokers ***P* < 0.01, **P* < 0.05. (ZIP 582 kb)
