# Supplementary material for: Differential impact of smoking on mortality and kidney transplantation among adult Men and Women undergoing dialysis
Source: BMC Nephrol. 2016 Jul 26;17:95. doi: 10.1186/s12882-016-0311-x (PMC4960807; doi:10.1186/s12882-016-0311-x)
Supplement: Additional file 1: Table S1. — Factors associated with the presence of Smoking at dialysis onset among new Dialysis Patients1. Multivariable analyses of factors associated with smoking at dialysis initiation. Model included demographic, clinical, biochemical, lifestyle, employment and functional status indicators measured at dialysis onset. C-statistic 78 %, 2 95 % confidence intervals. (DOC 59 kb) [file 12882_2016_311_MOESM1_ESM.doc]

## eTable 1: Factors associated with the presence of Smoking at dialysis onset among new Dialysis Patients¹

|  |  |  |
| --- | --- | --- |
| **Variable** |  | **Adjusted Odds Ratio (95% CI)²** |
|  |  |  |
|  |  |  |
| **Demographic** | |  |
| Age (years) | 18-40 | 9.79 (9.03-10.60) |
|  | 40-50 | 11.37 (10.54-12.27) |
|  | 50-60 | 8.48 (7.87-9.13) |
|  | 60-70 | 4.83 (4.49-5.19) |
|  | 70-80 | 2.41 (2.34-2.59) |
|  | >80 (referent) | 1.00 |
|  |  |  |
| Gender | Female (referent) | 1.00 |
|  | Male | 1.28 (1.15-1.32) |
|  |  |  |
| Race | White (referent) | 1.00 |
|  | Black | 0.79 (0.77-0.81) |
|  | Asian | 0.32 (0.29-0.36) |
|  | Native American | 0.81 (0.73-0.90) |
| **Comorbid** **Conditions** |  |  |
|  | Chronic lung disease (yes vs. no) | 4.94 (4.79-5.11) |
|  | Hypertension (yes vs no) | 1.73 (1.67-1.79) |
|  | Peripheral vascular disease (yes vs. no) | 1.59 (1.54-1.64) |
|  | Stroke (yes vs no) | 1.26 (1.21-1.31) |
|  | Coronary disease (yes vs. no) | 1.17 (1.13-1.20) |
|  | Malignancy (yes vs. no) | 1.15 (1.10-1.21) |
|  | Heart failure (yes vs. no) | 1.00 (0.97-1.03) |
| **Health indicators** |  |  |
|  | Body mass index (per Kg/m² increase) | 0.96 (0.96-0.96) |
|  | Serum albumin (per 10 g/L increase) | 0.96 (0.94-0.97) |
|  |  |  |
| **Kidney Function at Dialysis Initiation** | eGFR < 5 ml/min (referent) | 1.00 |
|  | eGFR 5-10 ml/min | 1.00 (0.98-1.02) |
|  | eGFR 10-15 ml/min | 0.97 (0.95-0.99) |
|  | eGFR > 15 ml/min | 0.96 (0.94-0.99) |
| **Lifestyle factors** |  |  |
|  | Alcohol dependence (yes vs. no) | 5.12 (4.85-5.39) |
|  | Drug dependence | 3.14 (2.95-3.35) |
| **Employment Status** |  |  |
|  | Unemployed (referent) | 1.00 |
|  | Full-time employment | 0.73 (0.70-0.77) |
|  | Part-time employment | 0.87 (0.79-0.95) |
|  | Homemaker | 0.83 (0.77-0.89) |
|  | Medical Leave of absence | 0.87 (0.82-0.93) |
|  | Retired due to age | 0.81 (0.78-0.85) |
|  | Retired due to disability | 1.02 (0.98-1.06) |
|  | Student | 0.29 (0.21-0.40) |
|  |  |  |
| **Functional Status** | Inability to transfer independently (yes vs. no) | 0.68 (0.60-0.76) |
|  | Inability to walk independently (yes vs. no) | 1.12 (1.04-1.19) |
|  |  |  |
| **Pre-dialysis care** | Erythropoietin use prior to dialysis (yes vs. no) | 0.85 (0.82-0.87) |
|  |  |  |

¹ Multivariable analyses of factors associated with smoking at dialysis initiation.

Model included demographic, clinical, biochemical, lifestyle, employment and functional status indicators measured at dialysis onset.

C-statistic 78%

² 95% confidence intervals,
