# Supplementary figures and images for: Differential impact of smoking on mortality and kidney transplantation among adult Men and Women undergoing dialysis
Source: BMC Nephrol. 2016 Jul 26;17:95. doi: 10.1186/s12882-016-0311-x (PMC4960807; doi:10.1186/s12882-016-0311-x)

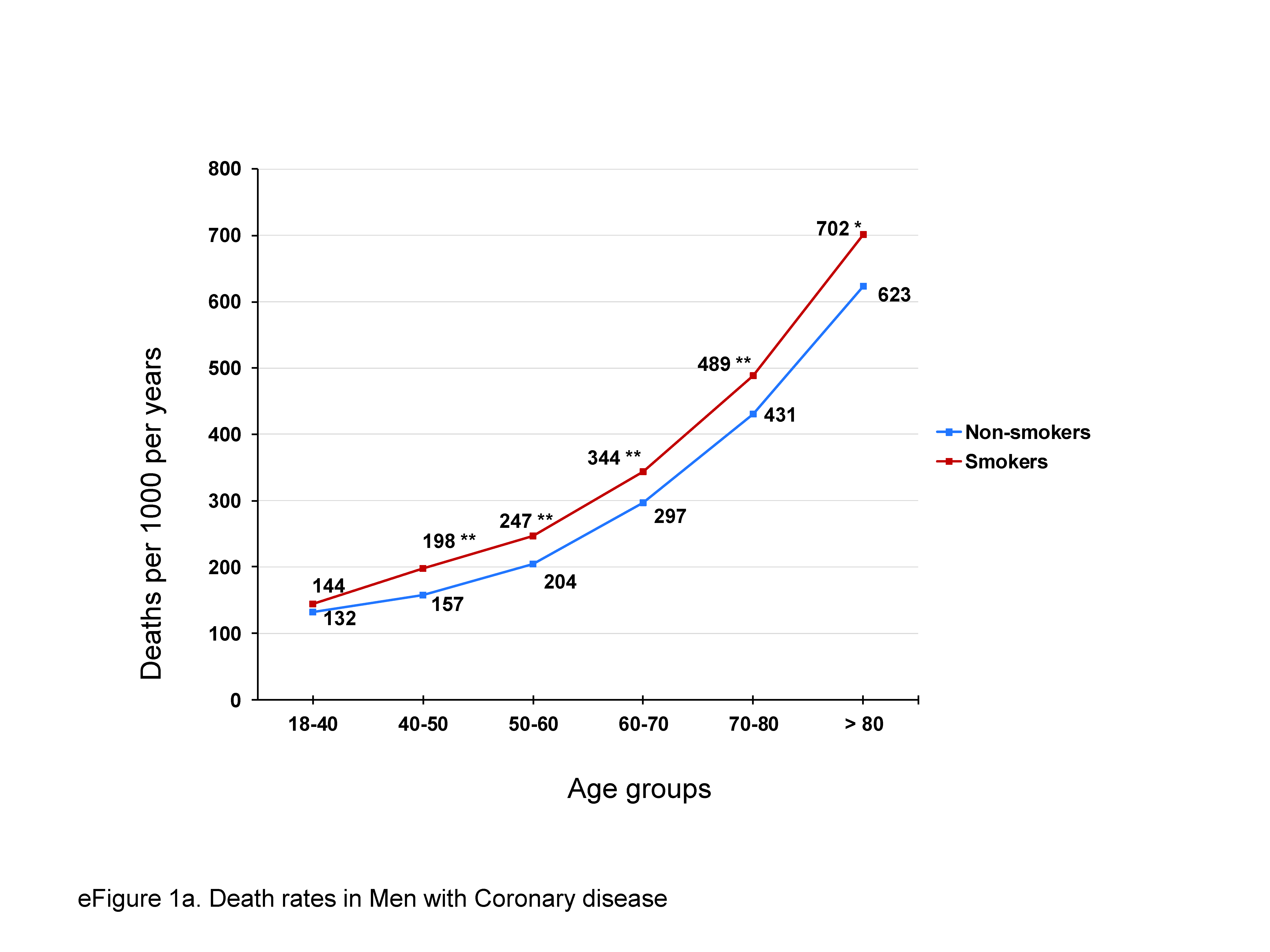

Supplement: Additional file 2: Figure S1a-c. — Age-specific mortality rates for Coronary disease, Peripheral Arterial Disease and Stroke for Men by smoking status. P–value for differences between smokers and non-smokers **P < 0.01, *P < 0.05. (ZIP 665 kb) [file 12882_2016_311_MOESM2_ESM.zip › figureS1/Supplemental-eFig 1aR3.tiff]

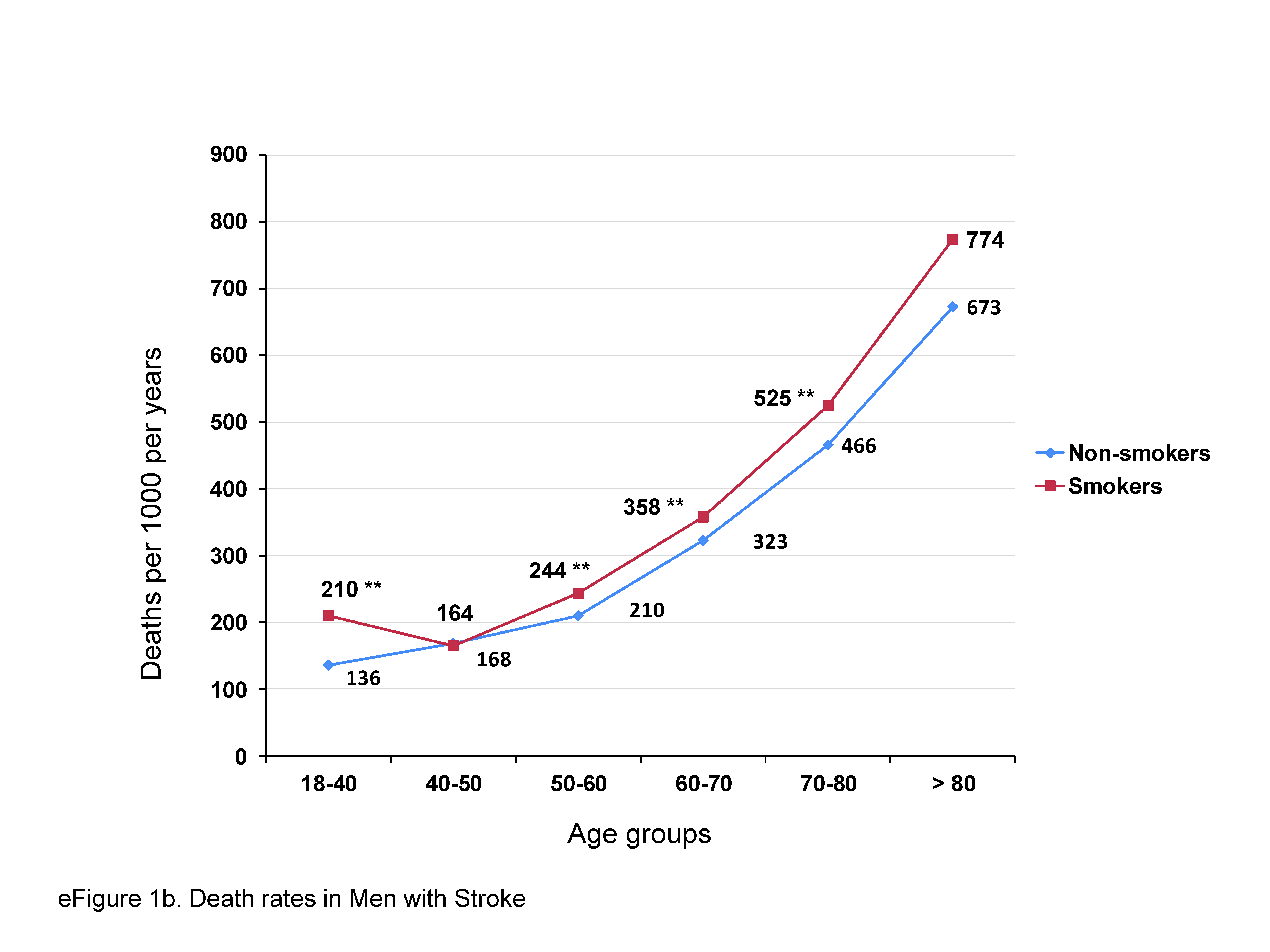

Supplement: Additional file 2: Figure S1a-c. — Age-specific mortality rates for Coronary disease, Peripheral Arterial Disease and Stroke for Men by smoking status. P–value for differences between smokers and non-smokers **P < 0.01, *P < 0.05. (ZIP 665 kb) [file 12882_2016_311_MOESM2_ESM.zip › figureS1/Supplemental-eFig 1bR3.tiff]

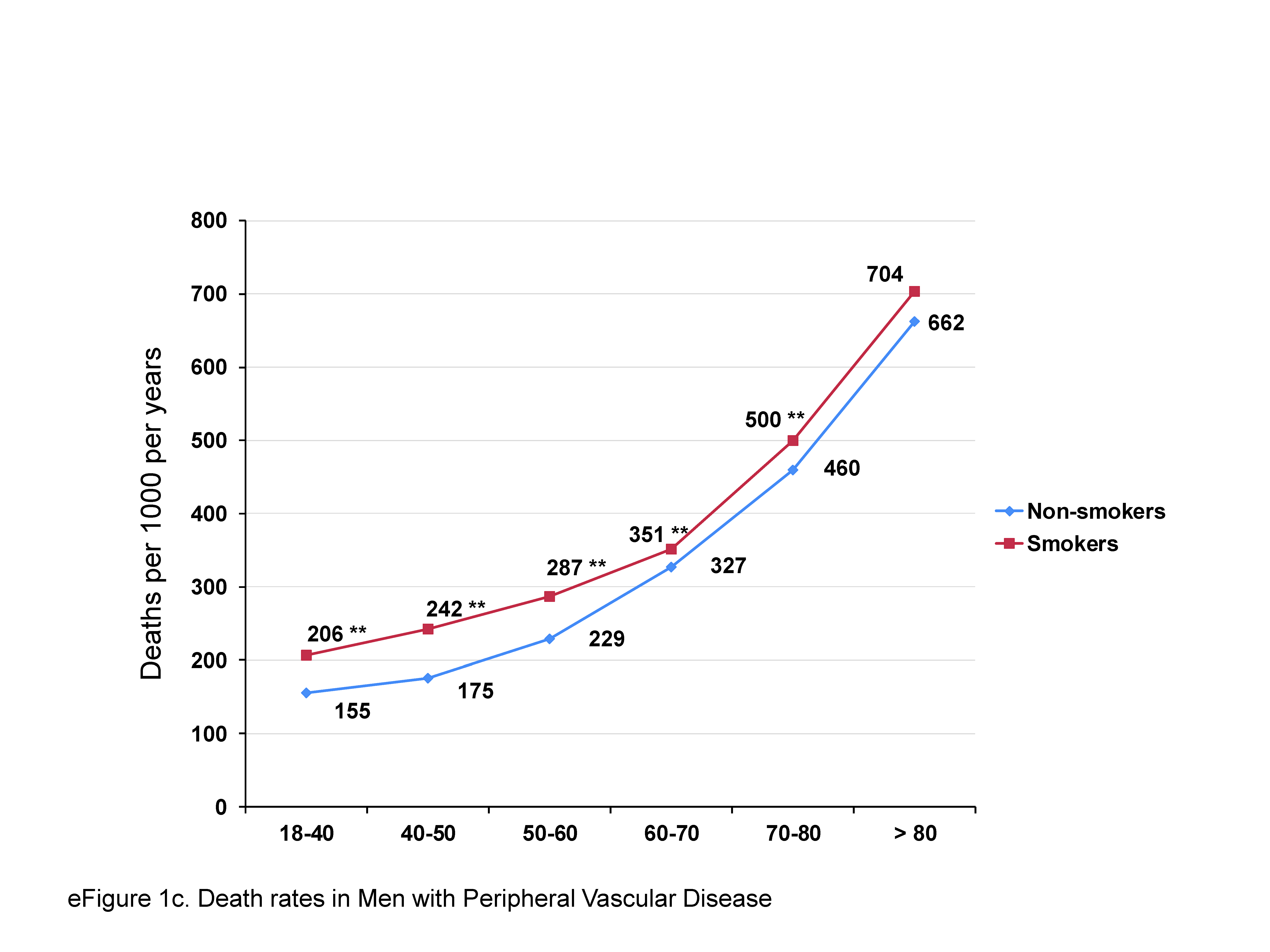

Supplement: Additional file 2: Figure S1a-c. — Age-specific mortality rates for Coronary disease, Peripheral Arterial Disease and Stroke for Men by smoking status. P–value for differences between smokers and non-smokers **P < 0.01, *P < 0.05. (ZIP 665 kb) [file 12882_2016_311_MOESM2_ESM.zip › figureS1/Supplemental-eFig 1cR3.tiff]

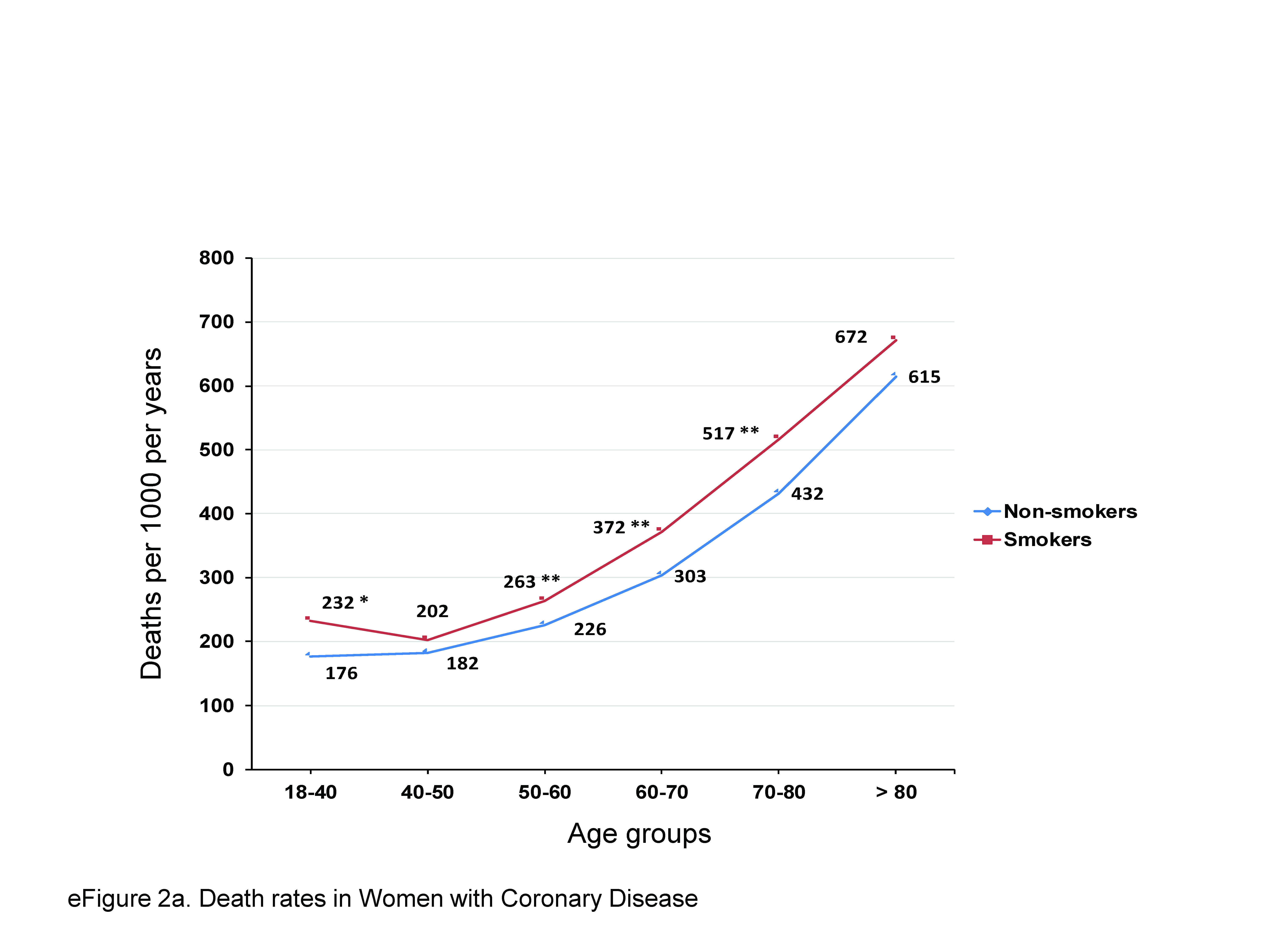

Supplement: Additional file 3: Figure S2a-c. — Age-specific mortality rates for Coronary disease, Peripheral Arterial Disease and Stroke for Women by smoking status. P–value for differences between smokers and non-smokers **P < 0.01, *P < 0.05. (ZIP 642 kb) [file 12882_2016_311_MOESM3_ESM.zip › FigureS2/Supplementary-eFig 2aR3.tiff]

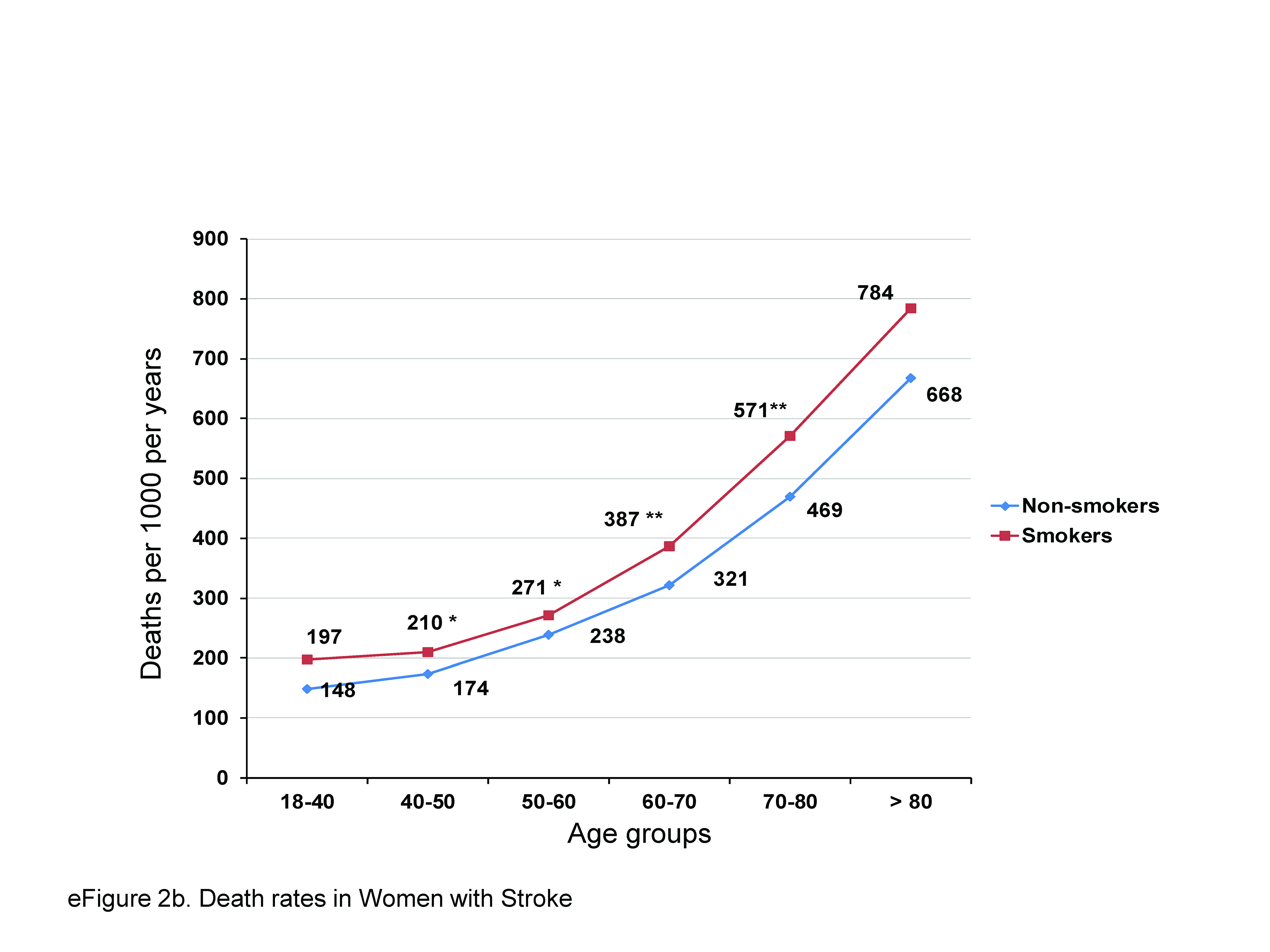

Supplement: Additional file 3: Figure S2a-c. — Age-specific mortality rates for Coronary disease, Peripheral Arterial Disease and Stroke for Women by smoking status. P–value for differences between smokers and non-smokers **P < 0.01, *P < 0.05. (ZIP 642 kb) [file 12882_2016_311_MOESM3_ESM.zip › FigureS2/Supplementary-eFig 2bR3.tiff]

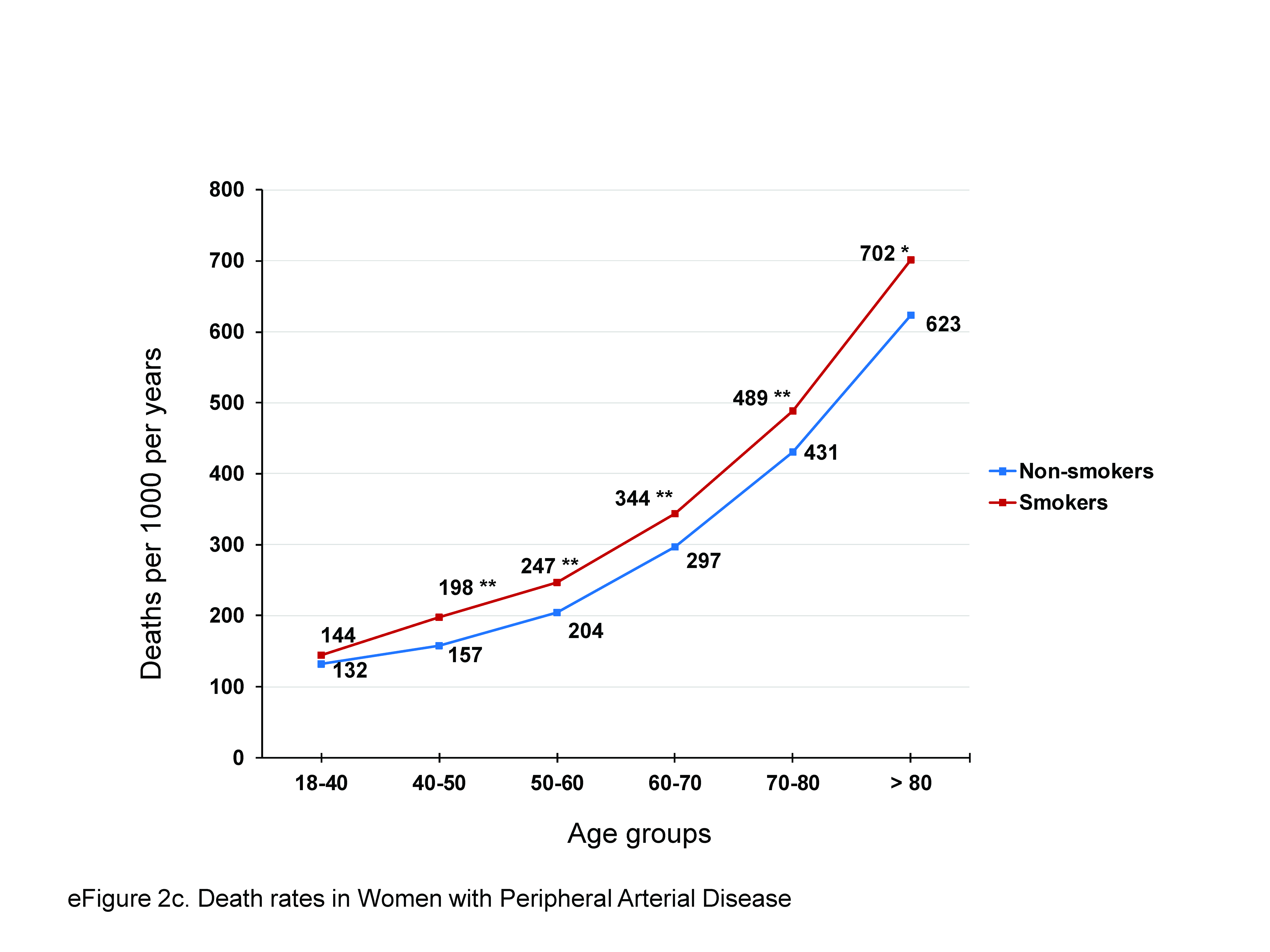

Supplement: Additional file 3: Figure S2a-c. — Age-specific mortality rates for Coronary disease, Peripheral Arterial Disease and Stroke for Women by smoking status. P–value for differences between smokers and non-smokers **P < 0.01, *P < 0.05. (ZIP 642 kb) [file 12882_2016_311_MOESM3_ESM.zip › FigureS2/Supplementary-eFig 2cR3.tiff]

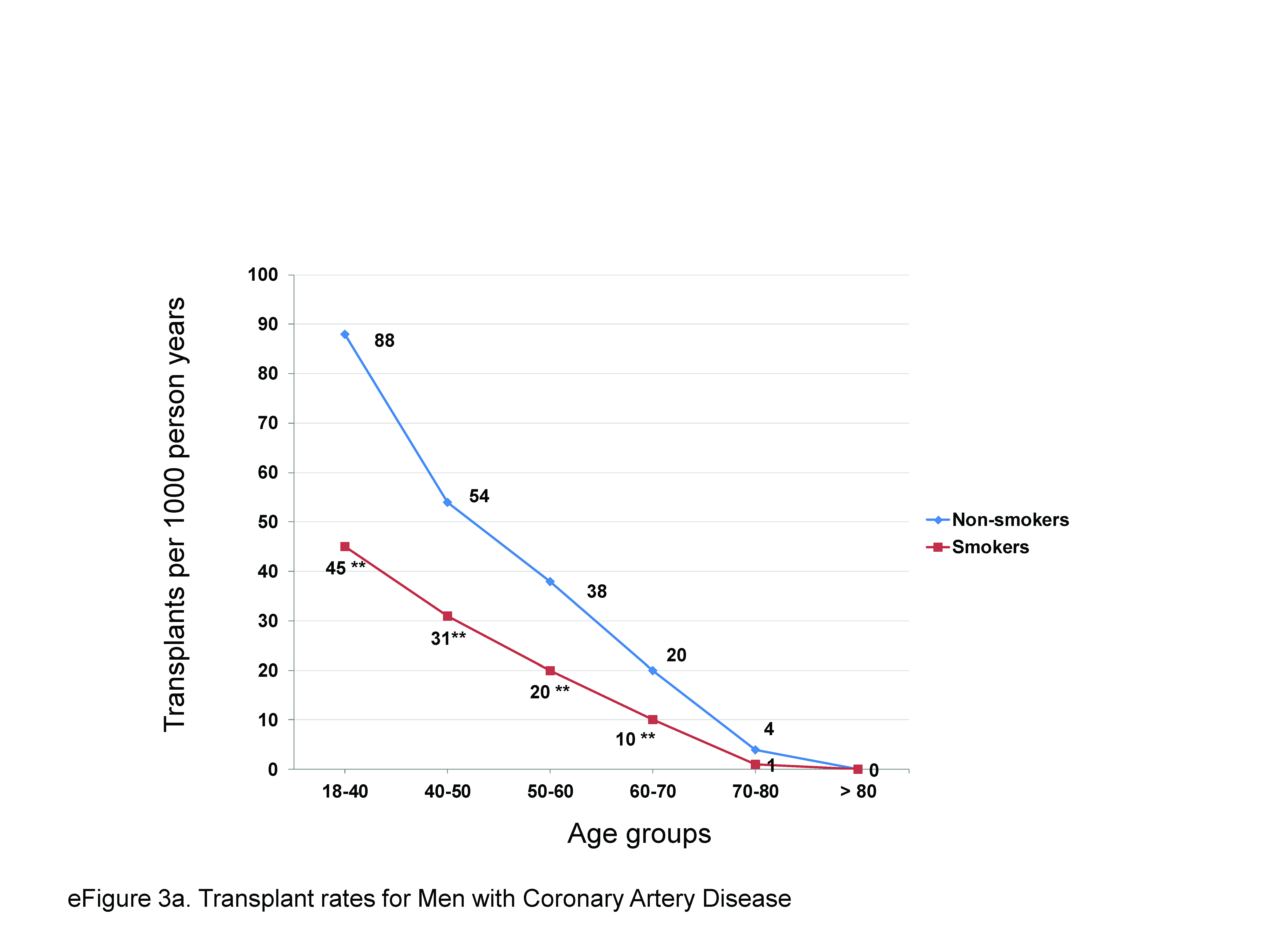

Supplement: Additional file 4: Figure S3a-c. — Age-specific transplantation rates for Coronary disease, Peripheral Arterial Disease and Stroke for Men by smoking status. P–value for differences between smokers and non-smokers **P < 0.01, *P < 0.05. (ZIP 586 kb) [file 12882_2016_311_MOESM4_ESM.zip › FigureS3/Supplementary eFig 3aR3.tiff]

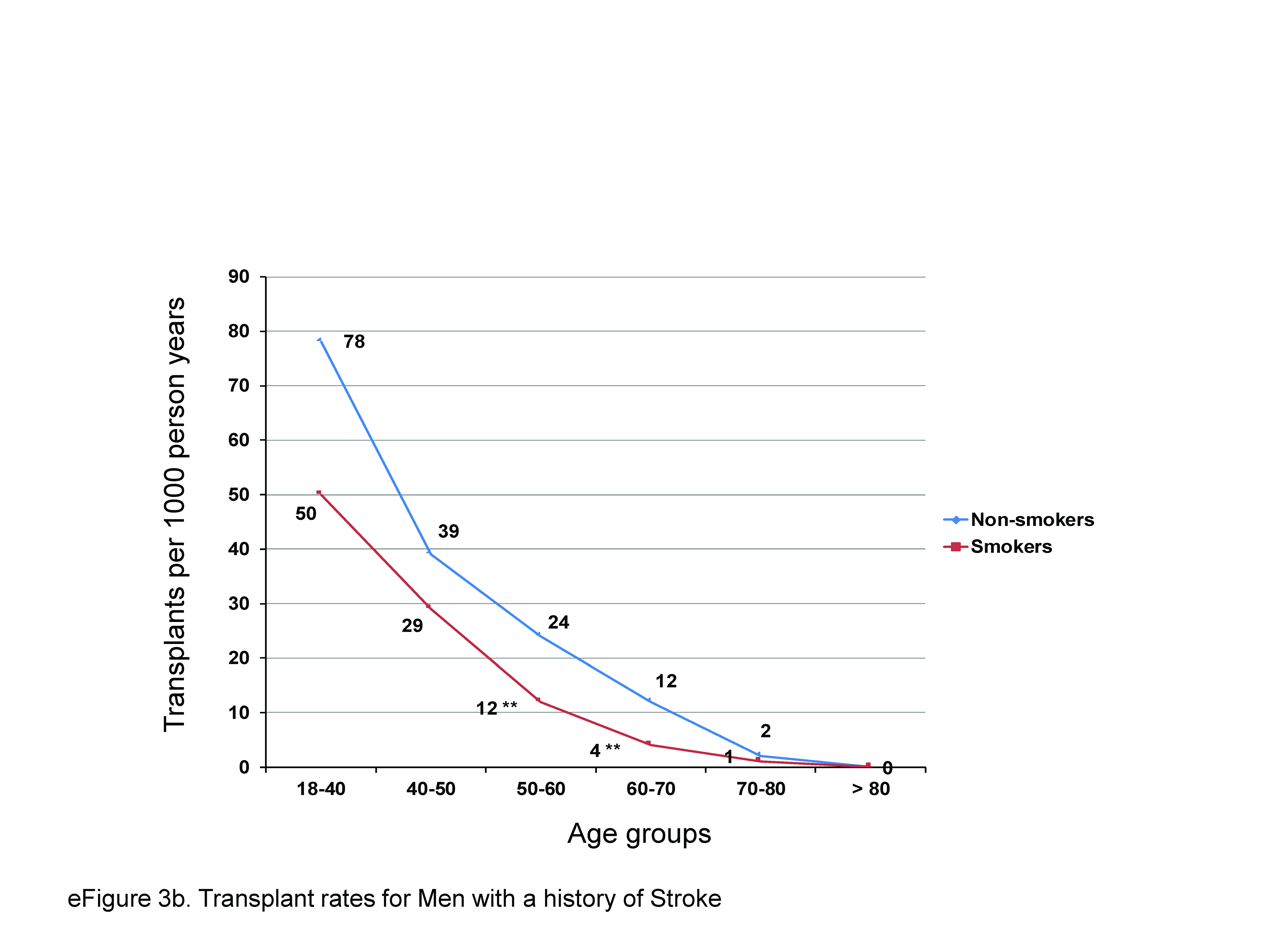

Supplement: Additional file 4: Figure S3a-c. — Age-specific transplantation rates for Coronary disease, Peripheral Arterial Disease and Stroke for Men by smoking status. P–value for differences between smokers and non-smokers **P < 0.01, *P < 0.05. (ZIP 586 kb) [file 12882_2016_311_MOESM4_ESM.zip › FigureS3/Supplementary eFig 3bR3.tiff]

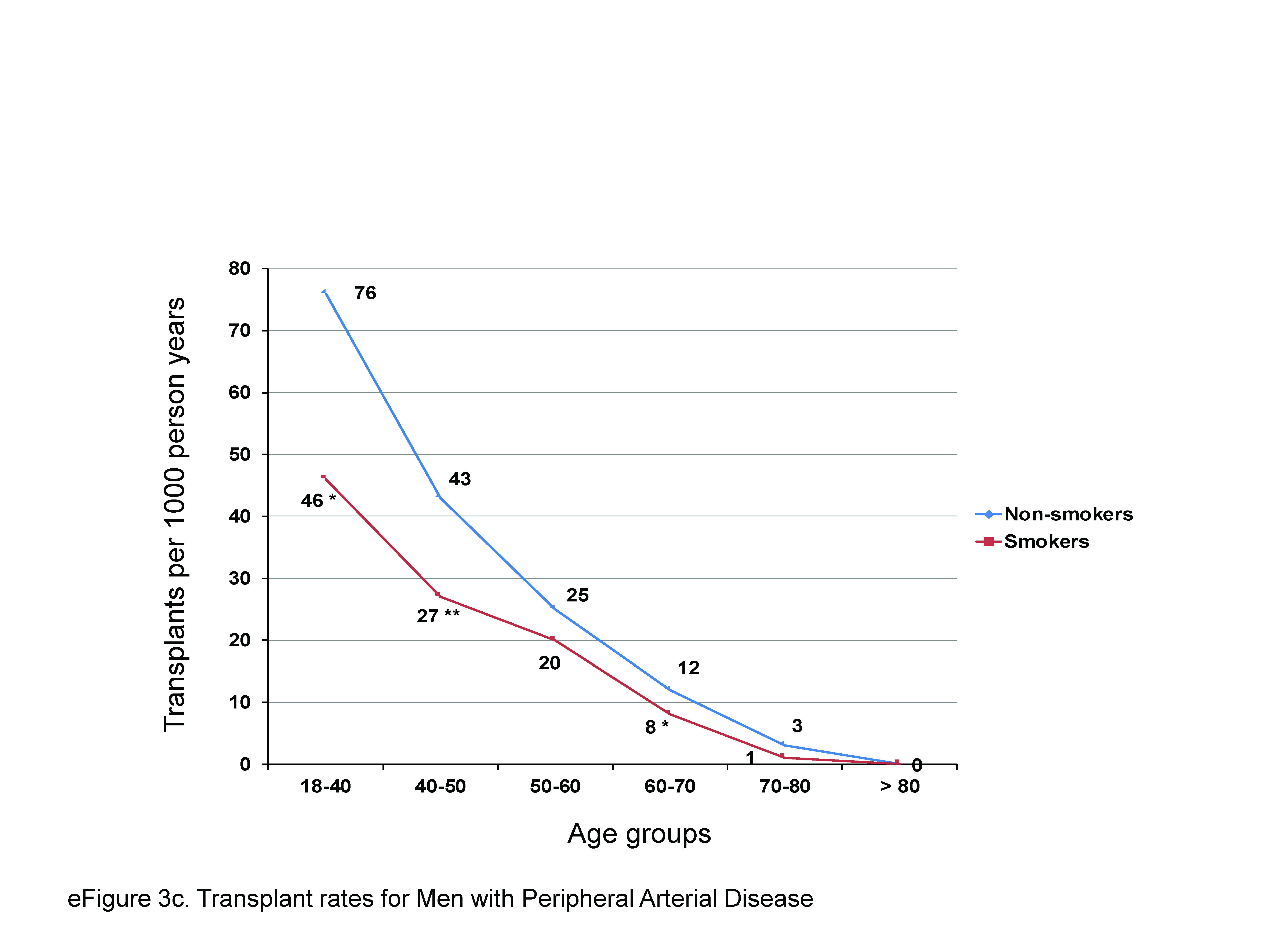

Supplement: Additional file 4: Figure S3a-c. — Age-specific transplantation rates for Coronary disease, Peripheral Arterial Disease and Stroke for Men by smoking status. P–value for differences between smokers and non-smokers **P < 0.01, *P < 0.05. (ZIP 586 kb) [file 12882_2016_311_MOESM4_ESM.zip › FigureS3/Supplementary eFig 3cR3.tiff]

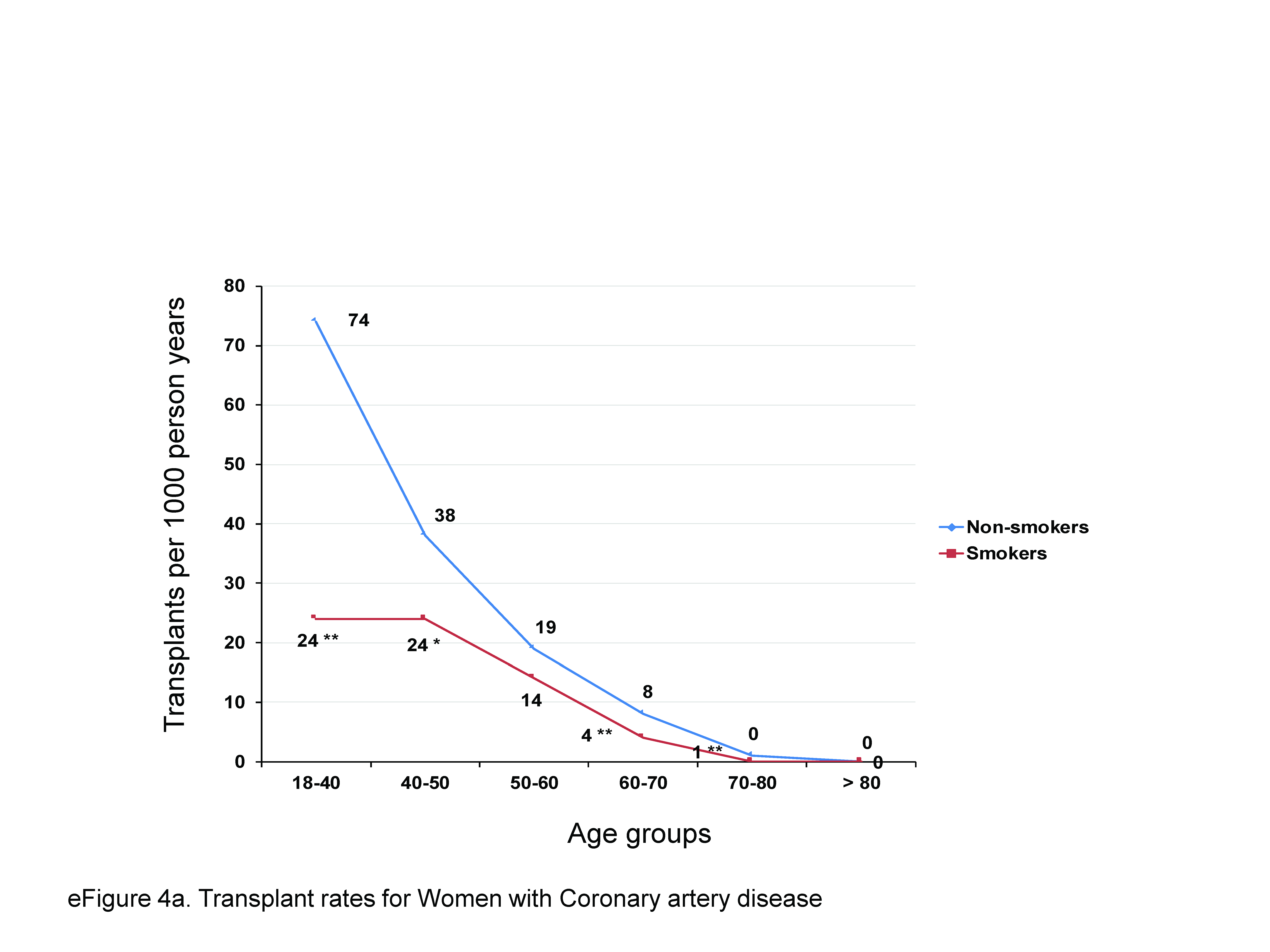

Supplement: Additional file 5: Figure S4a-c. — Age-specific transplantation rates for Coronary disease, Peripheral Arterial Disease and Stroke for Women by smoking status. P–value for differences between smokers and non-smokers **P < 0.01, *P < 0.05. (ZIP 582 kb) [file 12882_2016_311_MOESM5_ESM.zip › FigureS4/Supplementary eFig 4aR3.tiff]

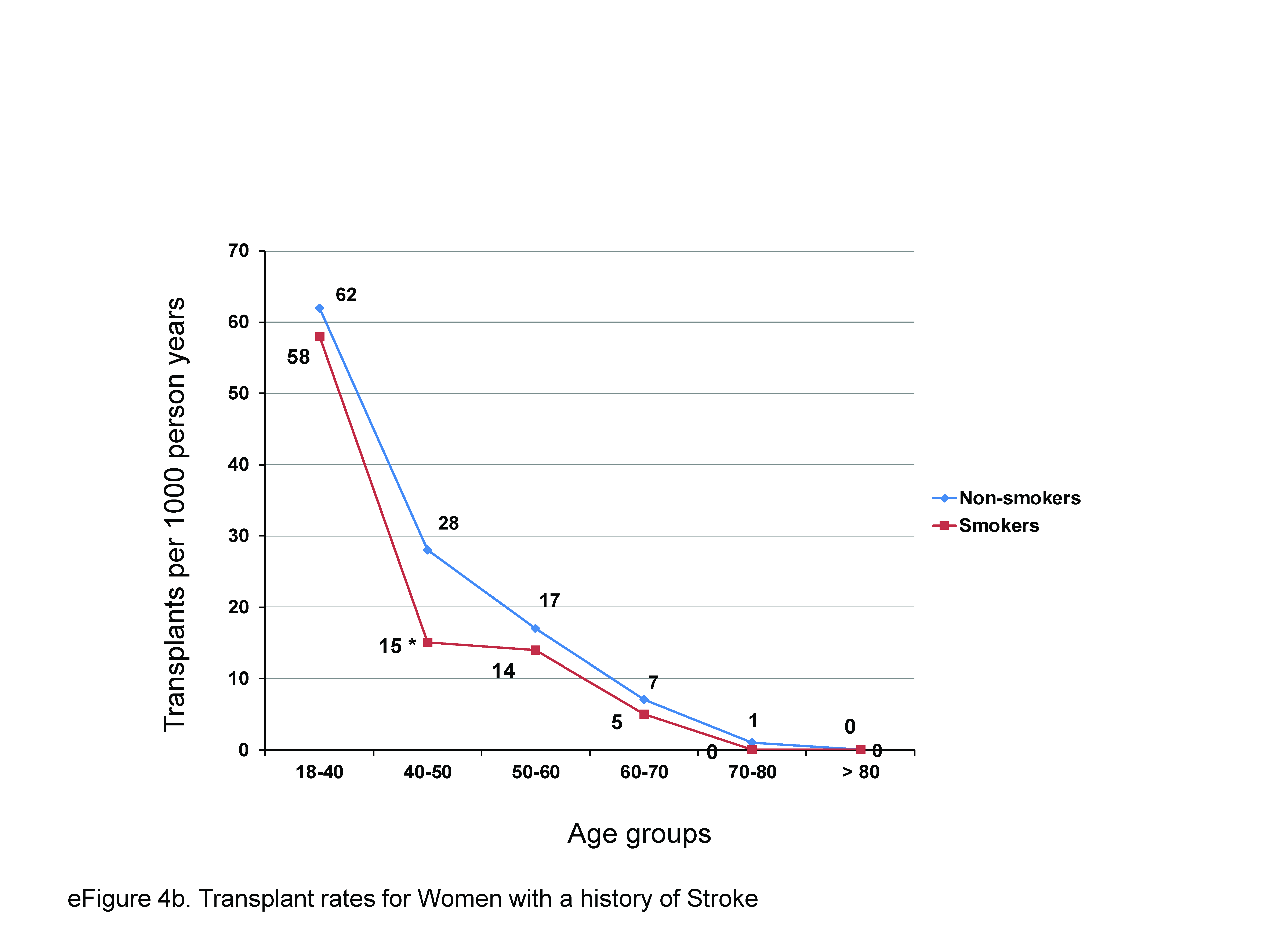

Supplement: Additional file 5: Figure S4a-c. — Age-specific transplantation rates for Coronary disease, Peripheral Arterial Disease and Stroke for Women by smoking status. P–value for differences between smokers and non-smokers **P < 0.01, *P < 0.05. (ZIP 582 kb) [file 12882_2016_311_MOESM5_ESM.zip › FigureS4/Supplementary eFig 4bR3.tiff]

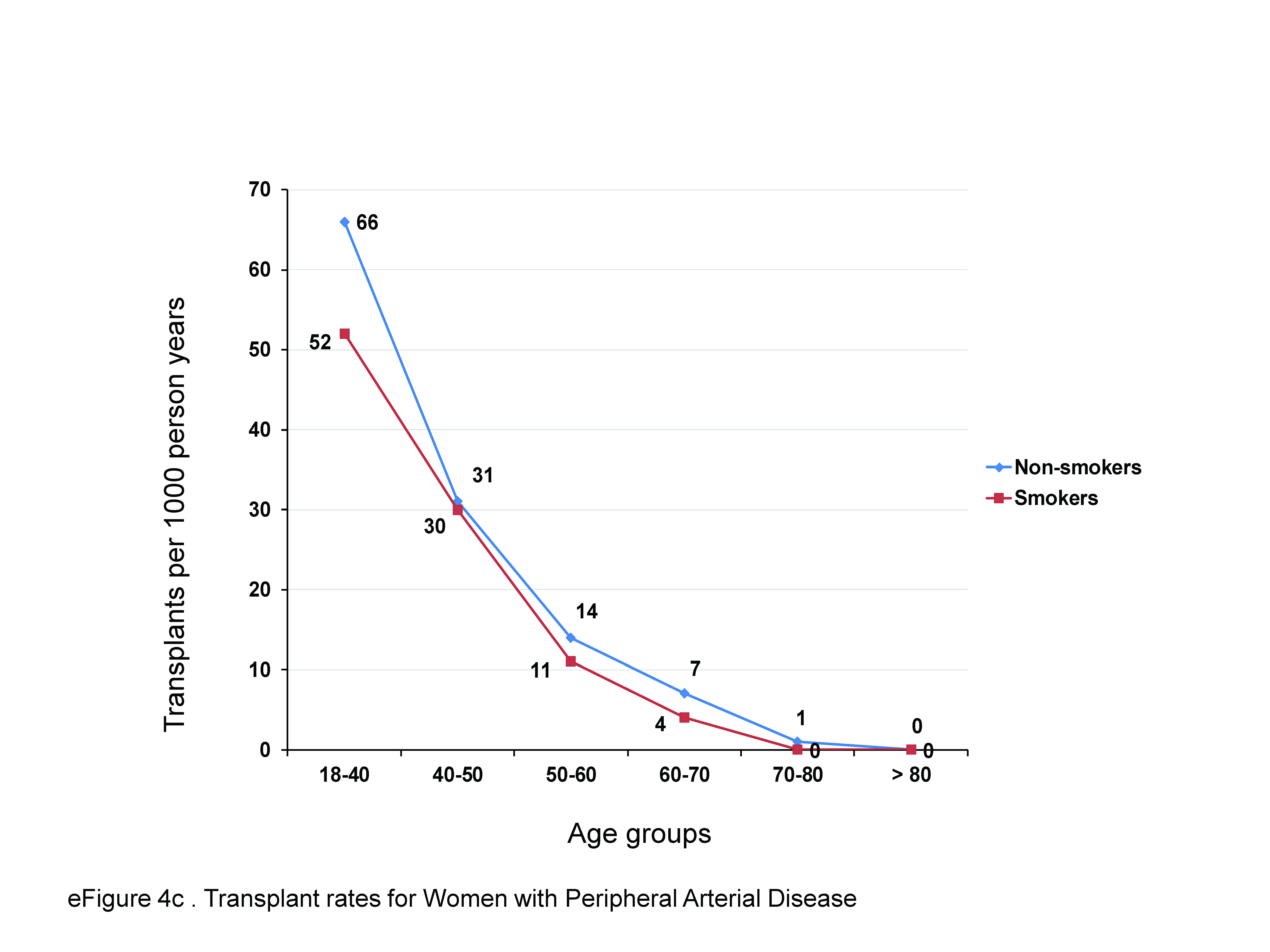

Supplement: Additional file 5: Figure S4a-c. — Age-specific transplantation rates for Coronary disease, Peripheral Arterial Disease and Stroke for Women by smoking status. P–value for differences between smokers and non-smokers **P < 0.01, *P < 0.05. (ZIP 582 kb) [file 12882_2016_311_MOESM5_ESM.zip › FigureS4/Supplementary eFig 4cR3.tiff]
